# Supplementary material for: Cancer exosomes induce tumor innervation
Source: Nat Commun. 2018 Oct 16;9:4284. doi: 10.1038/s41467-018-06640-0 (PMC6191452; doi:10.1038/s41467-018-06640-0)
Supplement: Supplementary file 1 — Supplementary Information [file 41467_2018_6640_MOESM1_ESM.pdf]

Cancer exosomes induce tumor innervation

Madeo et al.

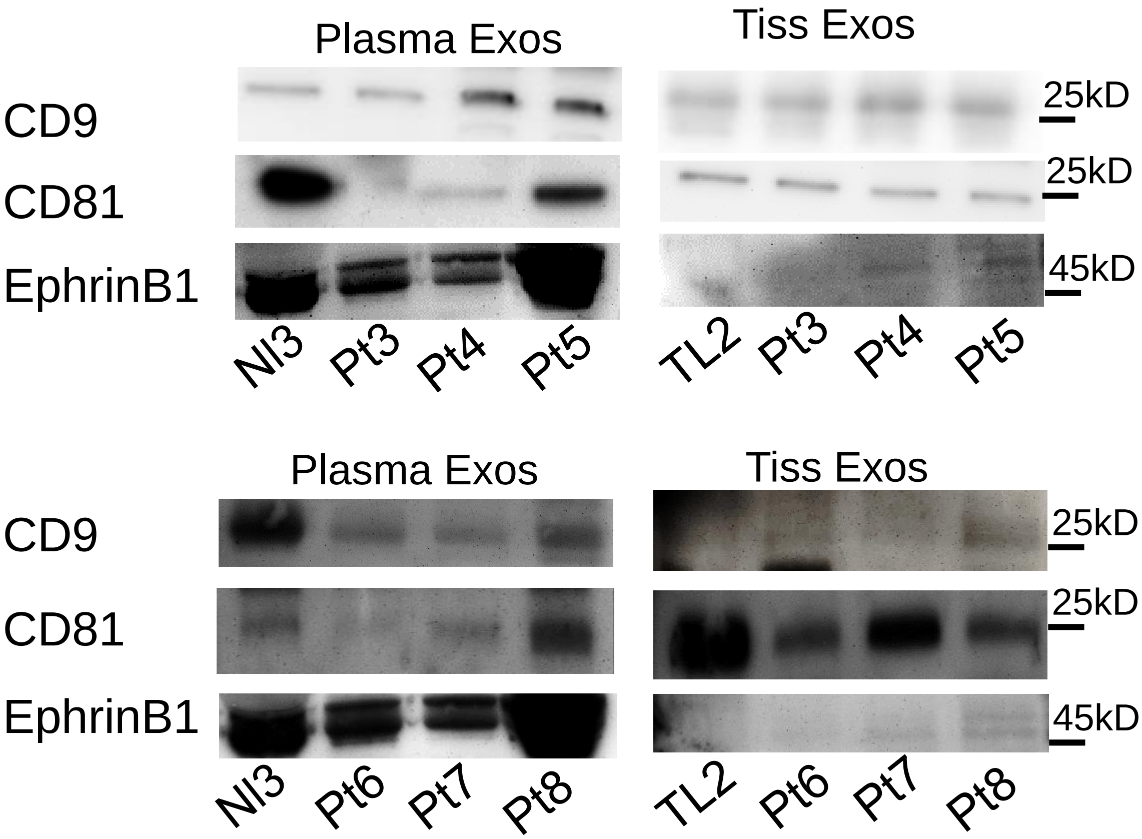

Supplementary Figure 1. Western blot analysis of human exosomes. Western blot analysis of exosomes (Exos) from human control (Nl), patient (Pt) plasma or matched tumor tissue. Control tissue is tonsil (TL). n=3 technical replicates with representative western blots shown.

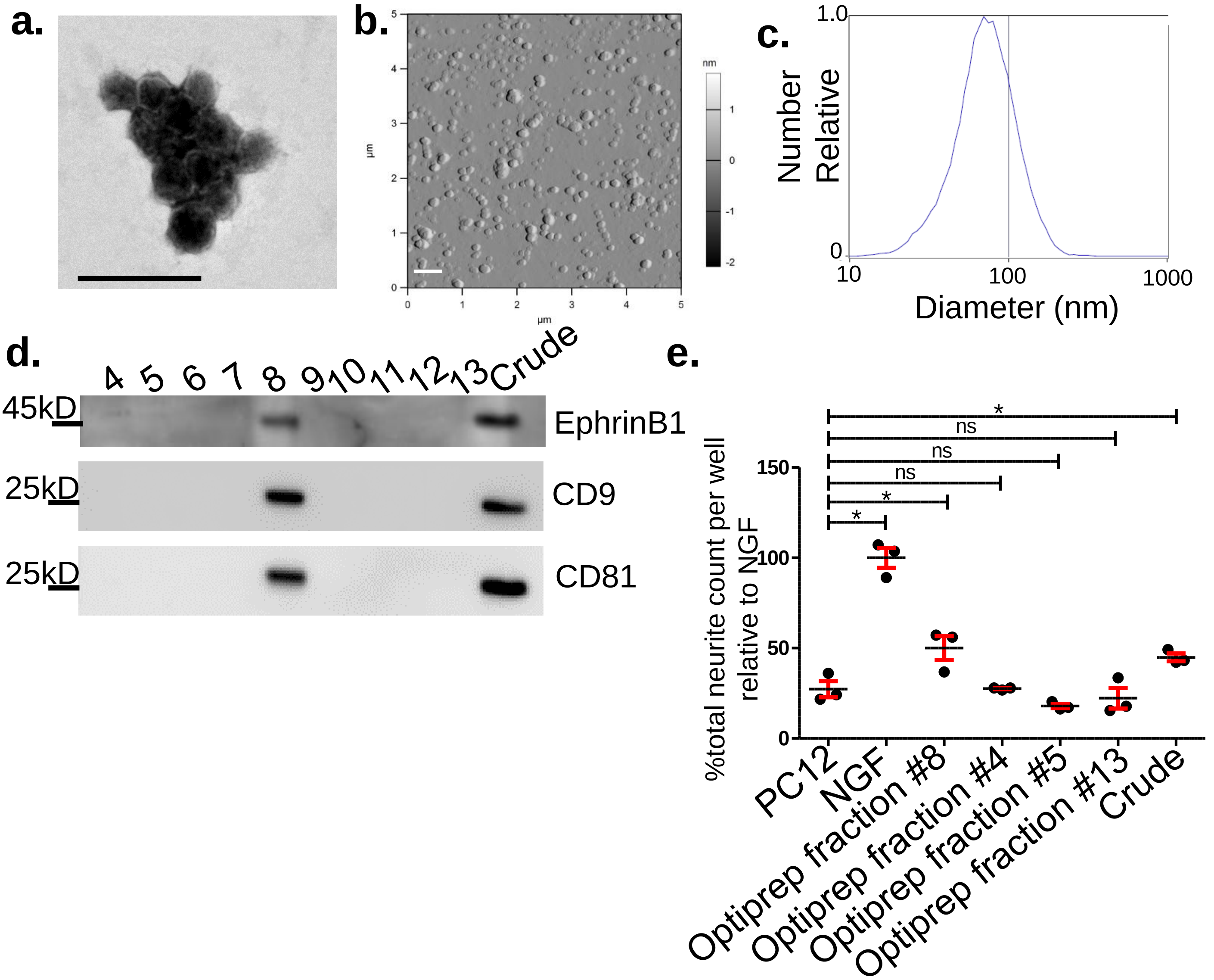

Supplementary Figure 2. Validation of mEERL exosome purification. A) Scanning electron micrograph of exosomes purified from mEERL parental conditioned media. Scale bar, 250 nm. n= 3 biological replicates. B) Atomic force microscopy amplitude trace of mEERL parental exosomes. Size range: 62-144 nm. Scale bar, 500 nm. n= 2 biological replicates. C) Nanoparticle tracking analysis showing size distribution of mEERL parental exosomes. n= 4 biological samples. D) Western blot analysis of mEERL *EphrinB1* exosomes purified by differential ultracentrifugation alone ("crude") or with optiprep density gradient centrifugation (fractions 4-13). n= 3 biological replicates. Experiment repeated n=2 times. E) Neurite outgrowth of exosomes from optiprep fractions 4, 5, 8, 13 and "crude" exosomes. NGF (100ng/ml) treatment serves as control. n=3 replicates/condition; experiment repeated twice. Statistical analysis by one-way ANOVA with post hoc Fisher's Least Significant Difference (LSD) test. LSD p values reported; *, $p < 0.01$; ns, not significant. All comparisons, LSD and Benjamini-Hochberg p values found in Supplemental Table 7. The variance between the groups statistically compared is similar. Central value used was the mean. Error bars, standard deviation.

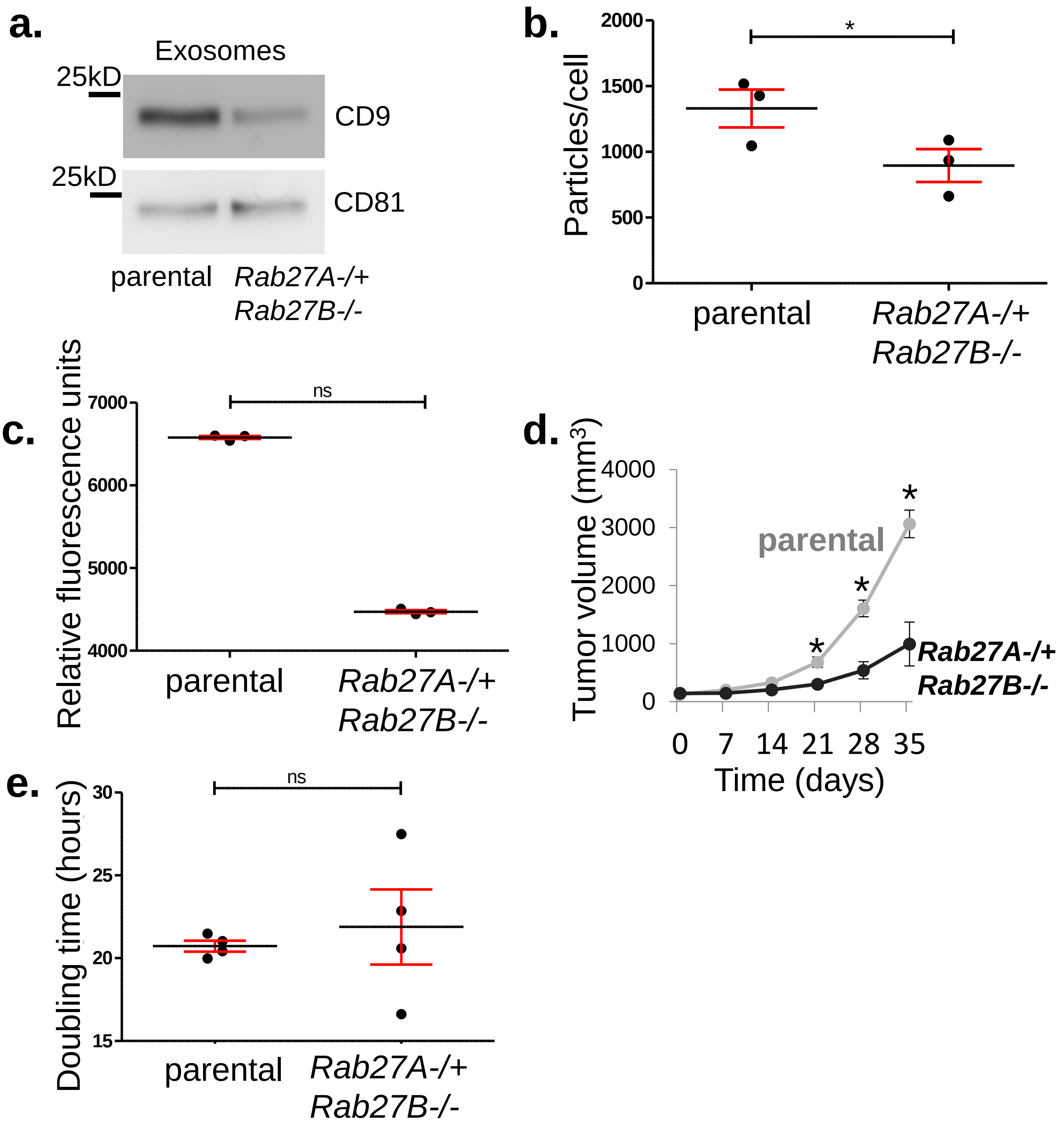

Supplementary Figure 3. mEERL tumors compromised in *Rab27A/B* have decreased exosome release. A) Western blot analysis of exosomes from the indicated sources; n=4 biological replicates. Exosomes were normalized to producing cell number. B) Exosome number by nanoparticle tracking analysis. Exosomes normalized to producing cell number; n=3 biological replicates. Statistical analysis by one-tailed student's t-test; *, p= 0.04. The variance between the groups statistically compared is similar. C) Representative relative fluorescence units of CFDA-SE labeled exosomes with technical replicates. Experiment was repeated n=4 times with biological replicates and generated similar results. Non-parametric test for significance indicates no difference in the means. ns, not significant. D) Tumor growth curves of mice implanted with mEERL parental (gray) or mEERL *Rab27A*$^{-/+}$*27B*$^{-/-}$ (black) tumors. n=7 mice/condition. Experiment performed one time. Statistical analysis by two-tailed student's t-test; *, p< 0.002. The variance between groups that compared is similar. E) Proliferation assay of the indicated cell lines, n=4 biological replicates with similar results. Statistical analysis by one-tailed student's t-test. The variance between the groups statistically compared is similar; ns, not significant. All error bars are standard error of the mean.

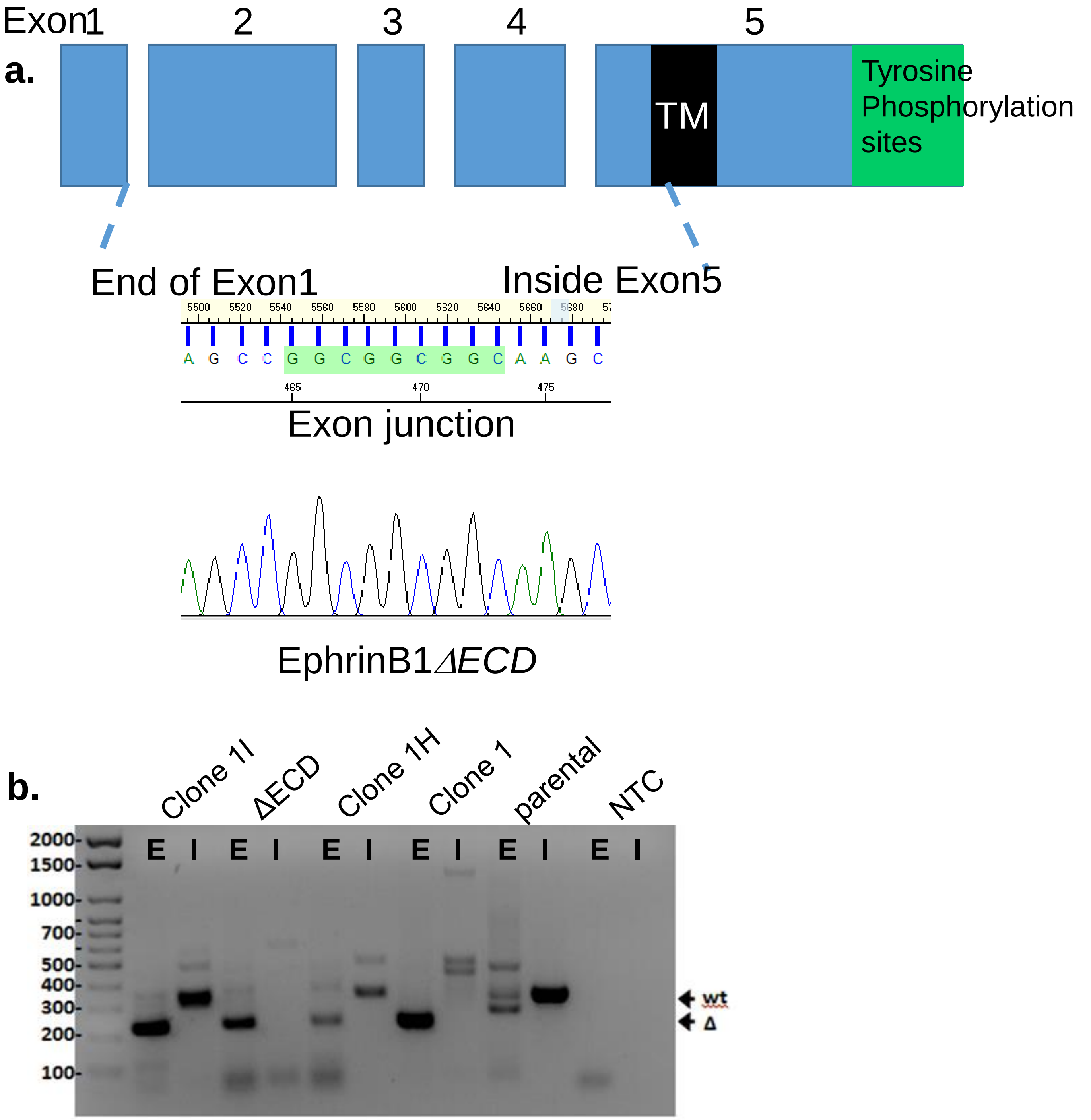

Supplementary Figure 4. Validation of mEERL *EphrinB1ΔECD*. A) Schematic of EphrinB1 exons showing the deletion in the *EphrinB1ΔECD* clone (dotted lines) which results in a junction between the end of exon 1 and within exon 5 (Exon Junction). The majority of the transmembrane domain (TM) domain is retained. Electropherogram of the exon junction is shown. B) PCR analysis of EphrinB1 CRISPR clones using external (E) and internal (I) primers. This CRISPR strategy leads to loss of amplification with internal probes when successful. Clones that lack amplification with internal primers were chosen for further validation; the mEERL *EphrinB1ΔECD* (ΔECD) clone was thus further validated. NTC, no template control. N= 3 biological replicates with similar results.

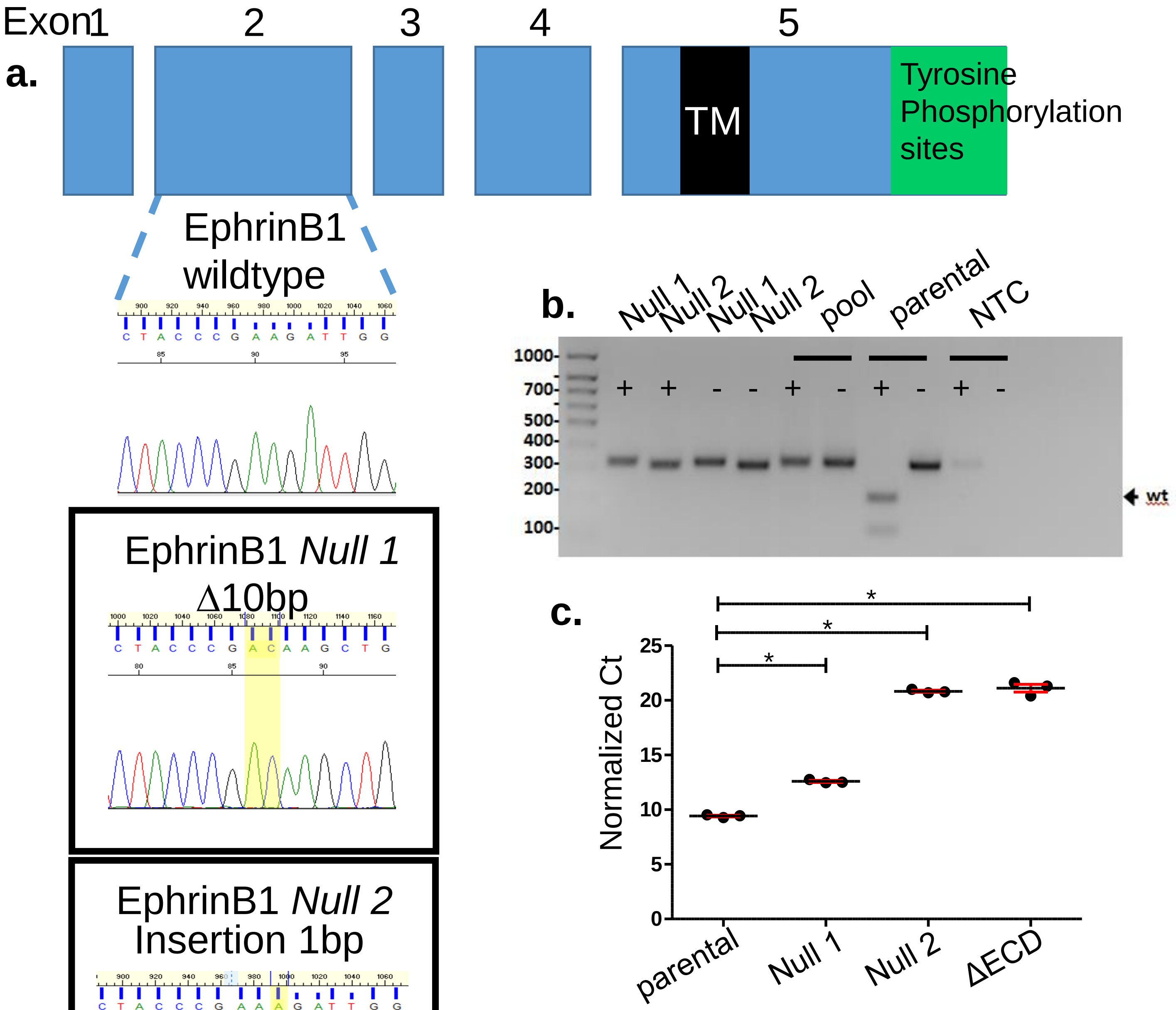

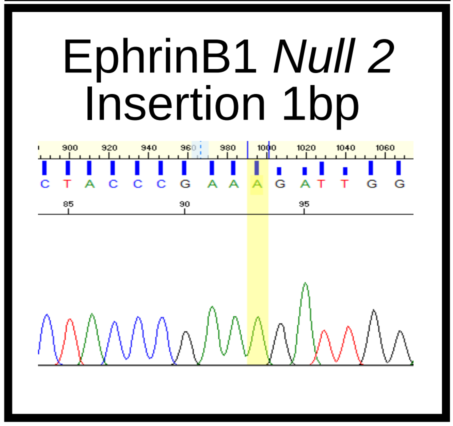

Supplementary Figure 5. Validation of mEERL EphrinB1 CRISPR *Null 1* and *Null 2*. A) Schematic of EphrinB1 exons and electropherograms of clones showing the alterations within exon 2 that generated the *Null 1* and *Null 2* clones which result in no EphrinB1 expression. The electropherogram for full length, wildtype EphrinB1 is also shown. B) This CRISPR strategy results in destruction of a restriction site if successful. mEERL parental cells served as a positive control. +, with restriction enzyme, -, without restriction enzyme. Pool refers to all clones pooled together. NTC, no template control. Wt, wildtype. C) Quantitative RT-PCR for EphrinB1 of CRISPR clones. Data normalized to beta actin. mEERL parental cells (parental) served as a positive control. Clones X, *Null 1*, *Null 2* and *ΔECD* were tested. N=3 biological replicates with similar results. Error bars, standard deviation.

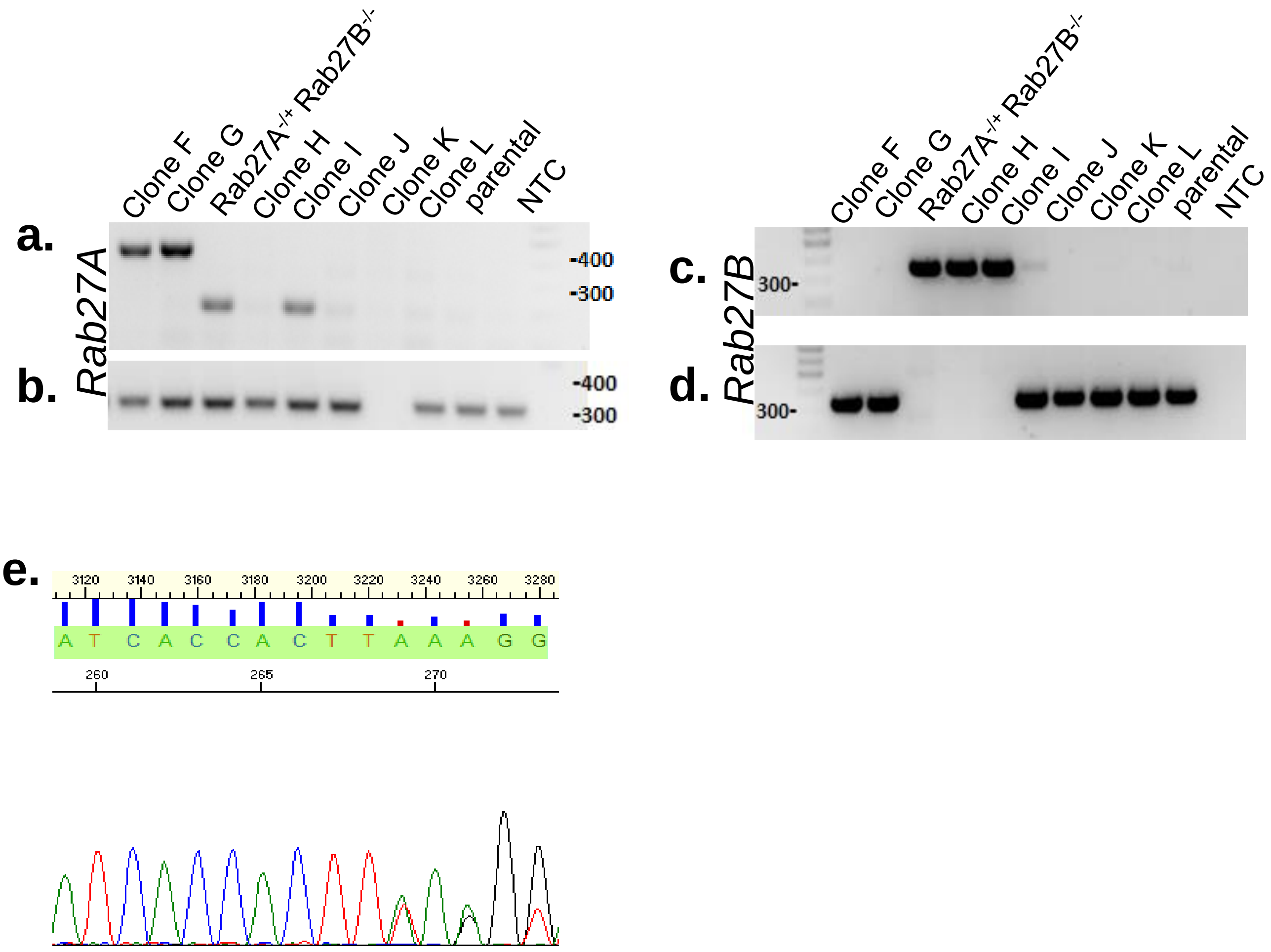

Supplementary Figure 6. Validation of mEERL *Rab27A-/+ Rab27B-/-*. Clones were screened using PCR primers distal to (A, C) or within (B,D) the expected regions of genomic deletion of *Rab27A* (A,B) and *Rab27B* (C, D). Clone *Rab27A-/+ Rab27B-/-* exhibited a larger than expected truncation of one *Rab27A* allele (A) but retained one wt allele (B) while *Rab27B* showed the predicted deletion product (C) and lack of wt template (D). n=3 biological replicates. E) Electropherogram from the mEERL *Rab27A-/+ Rab27B-/-* clone showing different repair mechanisms in the form of double peaks.

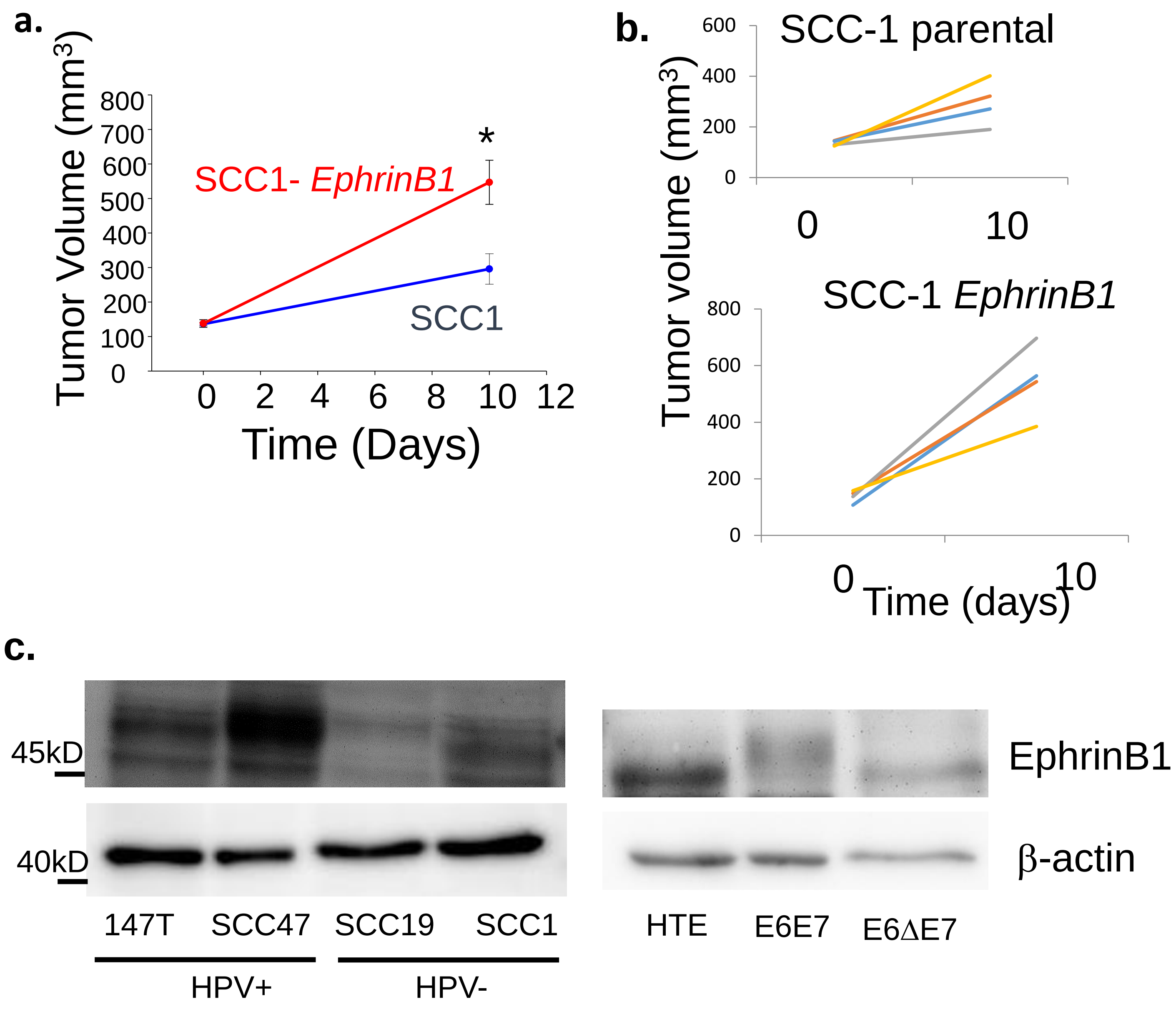

Supplementary Figure 7. EphrinB1 and tumor growth. A) Average tumor growth curves from mice (n=4/group) bearing SCC1 parental (blue line) or SCC1-*EphrinB1* (red line) tumors. Statistical test by two-tailed student's t-test; *, p= 0.009. The variance between groups compared is similar. Experiment performed one time. Central value used was the mean. Error bars are standard error of the mean. B) Individual tumor growth curves for mice in experiment in panel B. Each line represents one mouse. C) Western blot analysis of whole cell lysate from the indicated human cell lines.

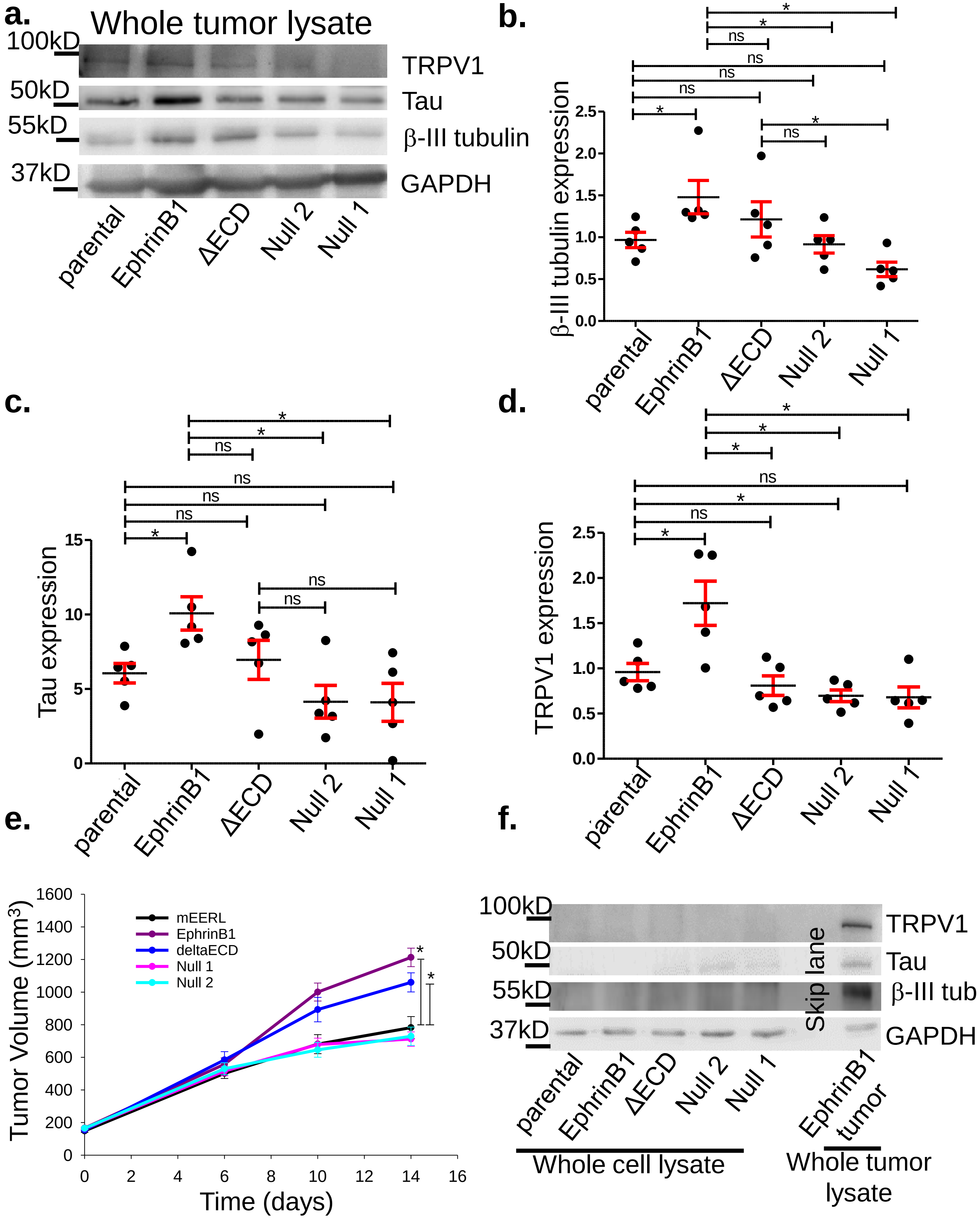

Supplementary Figure 8

Supplementary Figure 8. Increased EphrinB1 tumor expression potentiates tumor innervation *in vivo*. A) Western blot analysis of whole tumor lysates. Signals were normalized to GAPDH and densitometry was used to quantify innervation by: B) β-III tubulin, C) Tau and D) TRPV1 expression. n=5 tumors/condition were analyzed (i.e. 5 tumors/group were analyzed on 5 western blots, one representative western blot shown). Statistical analysis by one-way ANOVA with post hoc Fisher's Least Significant Difference (LSD) test; LSD p values reported; *, $p < 0.03$; ns, not significant. The variance between the groups statistically compared is similar. Central value used was the mean. n= 5 biological replicates/condition; experiment repeated two times. Error bars, standard deviation. All comparisons and LSD p values found in Supplemental Table 7 and 8. E) Tumor growth curves of mice (n=10 mice/group) bearing the indicated mEERL tumors. Statistical analysis by student's t-test; *, $p < 0.01$. Error bars are SEM. The variance between the groups statistically compared is similar. Central value used was the mean. F) Western blot analysis of whole cell lysates from the indicated cell lines and whole tumor lysate from mEERL *EphrinB1* tumor.

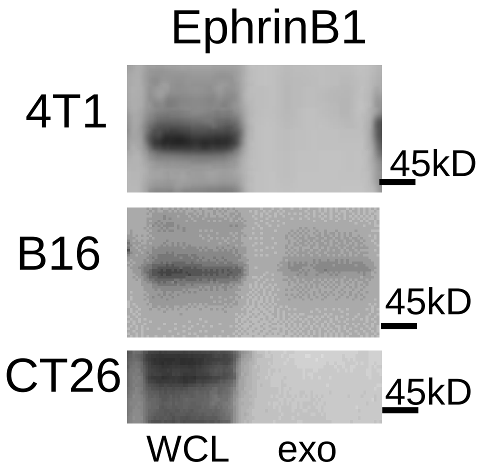

Supplementary Figure 9. Western blot analysis of whole cell lysate (WCL) and exosomes (exo) purified from the indicated cell lines.

Supplementary Table 1:Comparisons and LSD p values

Figure	comparisons	LSD p value
Figure 3A Top Panel	PC12 vs. NGF	0.0058
	PC12 vs. Nl1	0.3713
	PC12 vs. Nl2	0.7033
	PC12 vs. Pt1	0.0045
	PC12 vs. Pt2	0.0044
	NGF vs. Nl1	0.0183
	NGF vs. Nl2	0.0092
	NGF vs. Pt1	0.8199
	NGF vs. Pt2	0.8106
	Nl1 vs. Nl2	0.5916
	Nl1 vs. Pt1	0.0136
	Nl1 vs. Pt2	0.0134
	Nl2 vs. Pt1	0.007
	Nl2 vs. Pt2	0.0069
	Pt1 vs. Pt2	0.9904
Figure 3A Middle Panel	PC12 vs. NGF	< 0.0001
	PC12 vs. Nl1	0.4042
	PC12 vs. Nl2	0.537
	PC12 vs. Nl3	0.1364
	PC12 vs. Pt3	0.0009
	PC12 vs. Pt4	0.0314
	PC12 vs. Pt5	0.0114
	NGF vs. Nl1	< 0.0001
	NGF vs. Nl2	< 0.0001
	NGF vs. Nl3	< 0.0001
	NGF vs. Pt3	0.0004
	NGF vs. Pt4	< 0.0001
	NGF vs. Pt5	< 0.0001
	Nl1 vs. Nl2	0.8244
	Nl1 vs. Nl3	0.4887
	Nl1 vs. Pt3	0.006
	Nl1 vs. Pt4	0.1545
	Nl1 vs. Pt5	0.0643
	Nl2 vs. Nl3	0.3638
	Nl2 vs. Pt3	0.0037
	Nl2 vs. Pt4	0.1044
	Nl2 vs. Pt5	0.0416
	Nl3 vs. Pt3	0.0265
	Nl3 vs. Pt4	0.4448
	Nl3 vs. Pt5	0.2208
	Pt3 vs. Pt4	0.1179
	Pt3 vs. Pt5	0.2627
	Pt4 vs. Pt5	0.6314

Supplementary Table 2:Comparisons and LSD p values

Figure	comparisons	LSD p value
Figure 3A Bottom Panel	NGF vs. PC12	< 0.0001
	NGF vs. Nl1	< 0.0001
	NGF vs. Nl2	< 0.0001
	NGF vs. Nl3	< 0.0001
	NGF vs. Pt6	0.0019
	NGF vs. Pt7	0.0002
	NGF vs. Pt8	0.0001
	PC12 vs. Nl1	0.4027
	PC12 vs. Nl2	0.5356
	PC12 vs. Nl3	0.1352
	PC12 vs. Pt6	0.0002
	PC12 vs. Pt7	0.0022
	PC12 vs. Pt8d	0.0026
	Nl1 vs. Nl2	0.8239
	Nl1 vs. Nl3	0.4873
	Nl1 vs. Pt6	0.0013
	Nl1 vs. Pt7	0.0137
	Nl1 vs. Pt8	0.0164
	Nl2 vs. Nl3	0.3623
	Nl2 vs. pt6	0.0008
	Nl2 vs. Pt7	0.0085
	Nl2 vs. Pt8	0.0102
	Nl3 vs. Pt6	0.006
	Nl3 vs. Pt7	0.0572
	Nl3 vs. Pt8	0.0676
	Pt6 vs. Pt7	0.2862
	Pt6 vs. Pt8	0.2505
	Pt7 vs. Pt8	0.9305
Figure 3B Top panel	PC12 vs. NGF	0.0017
	PC12 vs. Pt1	0.0002
	PC12 vs. Pt2	0.001
	NGF vs. Pt1	0.0063
	NGF vs. Pt2	0.2685
	Pt1 vs. Pt2	0.0164

Supplementary Table 3:Comparisons and LSD p values

Figure	comparisons	LSD p value
Figure 3B Middle panel	NGF vs. PC12	< 0.0001
	NGF vs. TL2	< 0.0001
	NGF vs. Pt3	< 0.0001
	NGF vs. Pt4	< 0.0001
	NGF vs. Pt5	< 0.0001
	PC12 vs. TL2	0.2904
	PC12 vs. Pt3	0.0216
	PC12 vs. Pt4	0.0292
	PC12 vs. Pt5	0.0071
	TL2 vs. Pt3	0.1511
	TL2 vs. Pt4	0.1958
	TL2 vs. Pt5	0.0545
	Pt3 vs. Pt4	0.8729
	Pt3 vs. Pt5	0.5616
	Pt4 vs. Pt5	0.4617
Figure 3B Bottom panel	PC12 vs. NGF	< 0.0001
	PC12 vs. TL2	0.0001
	PC12 vs. Pt6	< 0.0001
	PC12 vs. Pt7	< 0.0001
	PC12 vs. Pt8	< 0.0001
	NGF vs. TL2	< 0.0001
	NGF vs. Pt6	0.9967
	NGF vs. Pt7	0.3217
	NGF vs. Pt8	0.0172
	TL2 vs. Pt6	< 0.0001
	TL2 vs. Pt7	0.0001
	TL2 vs. Pt8	0.0027
	Pt6 vs. Pt7	0.351
	Pt6 vs. Pt8	0.0239
	Pt7 vs. Pt8	0.1393

Supplementary Table 4:Comparisons and LSD p values

Figure	comparisons	LSD p value
Figure 5A	PC12 vs. NGF	< 0.0001
	PC12 vs. parental	0.0001
	PC12 vs. Rab27A/B	0.0115
	NGF vs. parental	0.0195
	NGF vs. Rab27A/B	0.0001
	parental vs. Rab27A/B	0.005
Figure 6A	Antibody:NGF vs. Antibody:Parental	0.3745
	Antibody:NGF vs. Antibody:EphrinB1	0.0009
	Antibody:NGF vs. NoAb:NGF	0.0019
	Antibody:NGF vs. NoAb:Parental	0.4863
	Antibody:NGF vs. NoAb:EphrinB1	0.0015
	Antibody:Parental vs. Antibody:EphrinB1	0.0047
	Antibody:Parental vs. NoAb:NGF	0.0102
	Antibody:Parental vs. NoAb:Parental	0.8417
	Antibody:Parental vs. NoAb:EphrinB1	0.0082
	Antibody:EphrinB1 vs. NoAb:NGF	0.6865
	Antibody:EphrinB1 vs. NoAb:Parental	0.0033
	Antibody:EphrinB1 vs. NoAb:EphrinB1	0.7739
	NoAb:NGF vs. NoAb:Parental	0.007
	NoAb:NGF vs. NoAb:EphrinB1	0.9067
	NoAb:Parental vs. NoAb:EphrinB1	0.0056

Supplementary Table 5:Comparisons and LSD p values

Figure	comparisons	LSD p value
Figure 6C	PC12 vs. NGF	< 0.0001
	PC12 vs. parental	0.0038
	PC12 vs. EphrinB1	< 0.0001
	PC12 vs. EphrinB1 DECD	0.092
	PC12 vs. null 1	0.0145
	PC12 vs. null 2	0.0011
	NGF vs. parental	< 0.0001
	NGF vs. EphrinB1	0.0054
	NGF vs. EphrinB1 DECD	< 0.0001
	NGF vs. null 1	< 0.0001
	NGF vs. null 2	< 0.0001
	parental vs. EphrinB1	0.0004
	parental vs. EphrinB1 DECD	0.1676
	parental vs. null 1	0.5824
	parental vs. null 2	0.639
	EphrinB1 vs. EphrinB1 DECD	< 0.0001
	EphrinB1 vs. null 1	< 0.0001
	EphrinB1 vs. null 2	0.0015
	EphrinB1 DECD vs. null 1	0.3968
	EphrinB1 DECD vs. null 2	0.069
	null 1 vs. null 2	0.3116
Figure 7A	PC12 vs. NGF	< 0.0001
	PC12 vs. SCC1 (-)	0.0253
	PC12 vs. SCC19 (-)	0.0016
	PC12 vs. 147T (+)	< 0.0001
	PC12 vs. SCC47 (+)	< 0.0001
	NGF vs. SCC1 (-)	< 0.0001
	NGF vs. SCC19 (-)	< 0.0001
	NGF vs. 147T (+)	< 0.0001
	NGF vs. SCC47 (+)	< 0.0001
	SCC1 (-) vs. SCC19 (-)	0.1618
	SCC1 (-) vs. 147T (+)	< 0.0001
	SCC1 (-) vs. SCC47 (+)	< 0.0001
	SCC19 (-) vs. 147T (+)	0.0003
	SCC19 (-) vs. SCC47 (+)	< 0.0001
	147T (+) vs. SCC47 (+)	0.0021

Supplementary Table 6:Comparisons and LSD p values

Figure	comparisons	LSD p value
Figure 7B	PC12 vs. NGF	< 0.0001
	PC12 vs. SCC1 (-)	0.0221
	PC12 vs. SCC1-EphrinB1	0.0001
	NGF vs. SCC1 (-)	< 0.0001
	NGF vs. SCC1-EphrinB1	< 0.0001
	SCC1 (-) vs. SCC1-EphrinB1	0.0031
Figure 7G	PC12 vs. NGF	< 0.0001
	PC12 vs. HTE	0.2314
	PC12 vs. HTE E6E7	< 0.0001
	PC12 vs. HTE E6delta/E7	0.0559
	NGF vs. HTE	< 0.0001
	NGF vs. HTE E6E7	< 0.0001
	NGF vs. HTE E6delta/E7	< 0.0001
	HTE vs. HTE E6E7	< 0.0001
	HTE vs. HTE E6delta/E7	0.3951
	HTE E6E7 vs. HTE E6delta/E7	0.0002
Figure 8B	NGF vs. PC12	0.0017
	NGF vs. CT26	0.2579
	NGF vs. B16	0.3318
	NGF vs. 4T1	0.2963
	PC12 vs. CT26	0.0125
	PC12 vs. B16	0.0004
	PC12 vs. 4T1	0.0106
	CT26 vs. B16	0.0507
	CT26 vs. 4T1	0.924
	B16 vs. 4T1	0.0598

Supplementary Table 7:Comparisons and LSD p values

Figure	comparisons	LSD p value
Suppl Figure 2E	PC12 vs. NGF	< 0.0001
	PC12 vs. optiprep fraction #8	0.0025
	PC12 vs. optiprep fraction #4	0.9669
	PC12 vs. optiprep fraction #5	0.1486
	PC12 vs. optiprep fraction #13	0.4279
	PC12 vs. Crude EVs	0.013
	NGF vs. optiprep fraction #8	< 0.0001
	NGF vs. optiprep fraction #4	< 0.0001
	NGF vs. optiprep fraction #5	< 0.0001
	NGF vs. optiprep fraction #13	< 0.0001
	NGF vs. Crude EVs	< 0.0001
	optiprep fraction #8 vs. optiprep fraction #4	0.0027
	optiprep fraction #8 vs. optiprep fraction #5	0.0001
	optiprep fraction #8 vs. optiprep fraction #13	0.0005
	optiprep fraction #8 vs. Crude EVs	0.4156
	optiprep fraction #4 vs. optiprep fraction #5	0.1385
	optiprep fraction #4 vs. optiprep fraction #13	0.405
	optiprep fraction #4 vs. Crude EVs	0.0141
	optiprep fraction #5 vs. optiprep fraction #13	0.488
	optiprep fraction #5 vs. Crude EVs	0.0006
	optiprep fraction #13 vs. Crude EVs	0.0026
Suppl Figure 8B	parental vs. EphrinB1	0.0249
	parental vs. DECD	0.2565
	parental vs. null2	0.8044
	parental vs. null1	0.1107
	EphrinB1 vs. DECD	0.2229
	EphrinB1 vs. null2	0.0145
	EphrinB1 vs. null1	0.0006
	DECD vs. null2	0.1713
	DECD vs. null1	0.0102
	null2 vs. null1	0.1716

Supplementary Table 8:Comparisons and LSD p values

Figure	comparisons	LSD p value
Suppl Figure 8C	parental vs. EphrinB1	0.0196
	parental vs. DECD	0.5776
	parental vs. null2	0.2406
	parental vs. null1	0.232
	EphrinB1 vs. DECD	0.0628
	EphrinB1 vs. null2	0.0013
	EphrinB1 vs. null1	0.0012
	DECD vs. null2	0.091
	DECD vs. null1	0.0872
	null2 vs. null1	0.9818
Suppl Figure 8D	parental vs. EphrinB1	0.001
	parental vs. DECD	0.4582
	parental vs. null2	0.2007
	parental vs. null1	0.1738
	EphrinB1 vs. DECD	0.0002
	EphrinB1 vs. null2	< 0.0001
	EphrinB1 vs. null1	< 0.0001
	DECD vs. null2	0.5772
	DECD vs. null1	0.5207
	null2 vs. null1	0.9315

Supplementary Table 9: References for antibodies used for western blot analysis

Antibody	Reference
1) CD9 (Abcam, ab92726)	Coulter ME, et al *Cell Rep*. 24: 973-986 (2018)
2) CD81(sc-166029, clone B-11)	Navakanitworakul et al *Sci Rep*. 6: 25486 (2016)
3) EphrinB1 (R&D sys AF473)	Dong, LD et al *J Neurosci* 35: 5409-21 (2015)
4) EphrinB1 (LSBio LS-C108001)	Colbert, PL et al *Oncotarget* 6: 953-68 (2015)
5) β-III tubulin (Abcam, ab18207)	Ruibin W et al *Biomed Pharma* 100:349-57 (2018)
6) GAPDH (Thermo, AM4300)	Hunter MR, et al *Biochem J* 474: 3615-26 (2017)
7) β actin (Sigma, A2228)	Jin, R et al *J Cell Sci* 127: 3116-30 (2014)
8) Tau (Abcam, ab75714)	Wu, J et al *Oncotarget* 8: 40843-56 (2017)
9) P-Erk1/2 (Millipore, ABS44)	Frank, D et al *MBio* 9: pii e00782-18 (2018)
10) TRPV1 (Novus, NB100-98897)	Issa A, et al *Neurosci Lett* 519: 26-30 (2012)<br>Chen J, et al Cardio Diabet 14: 22-35 (2015).

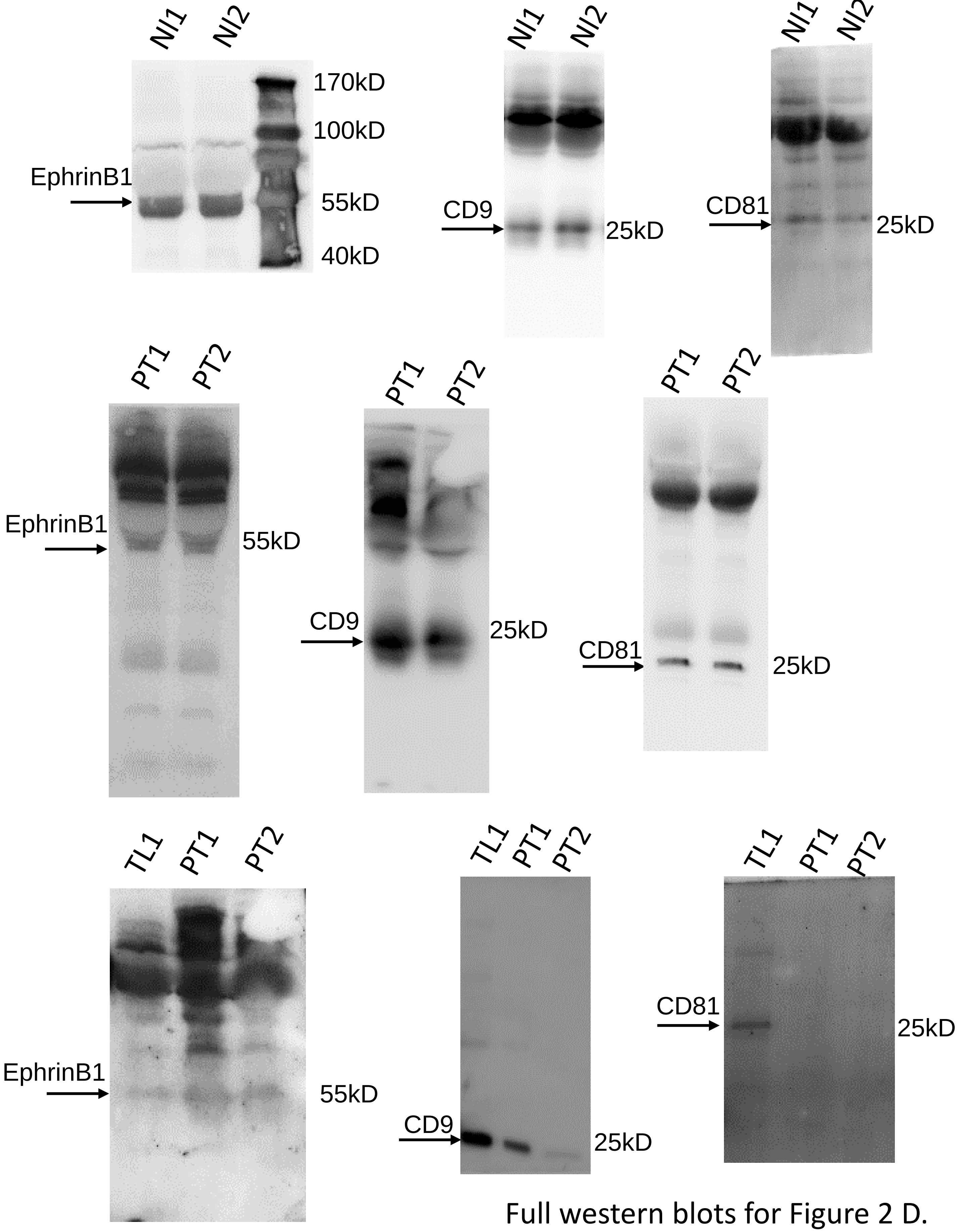

Full western blots for Figure 2 D.

Supplementary Figure 10

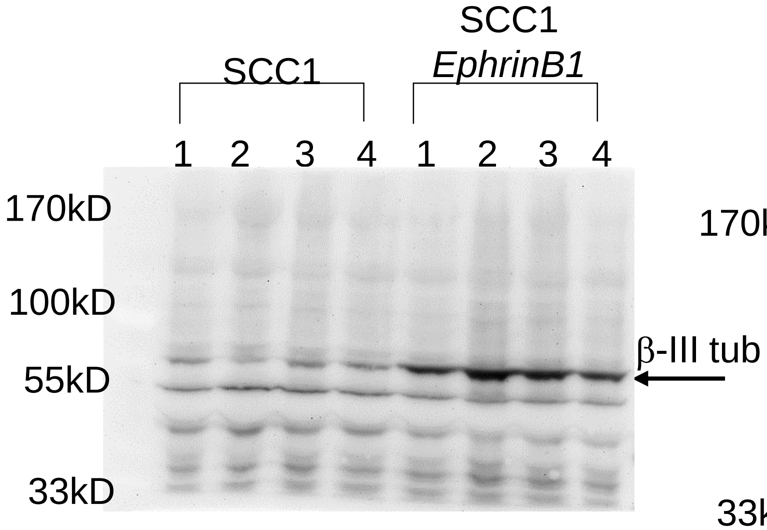

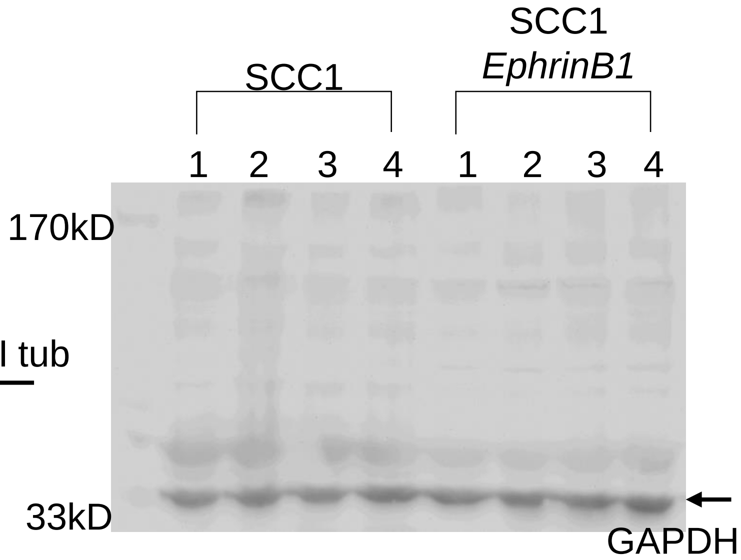

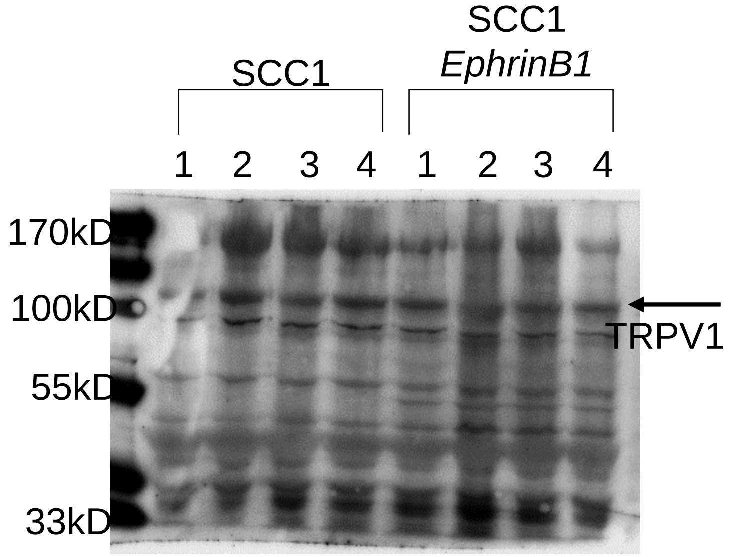

Full western blots for Figure 7 C.

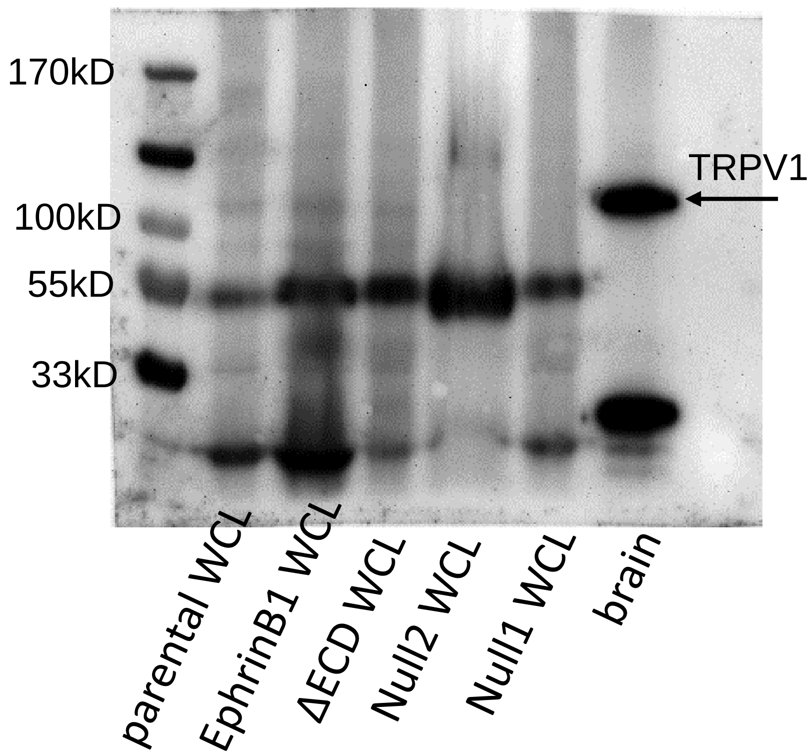

For reference, an additional western in which whole brain lysate was run as a control is included for validation of the TRPV1 band. The samples included with this western blot are whole cell lysates (WCL) from the indicated cell lines. The predicted molecular weight of TRPV1 is approximately 100kD. This is validated in a publication by Chen et al where the 100kD TRPV1 band is eliminated in tissue lysate from TRPV1 knockout mice (*Cardiovascular Diabetology* 14: 22-35 (2015).

Supplementary Figure 11

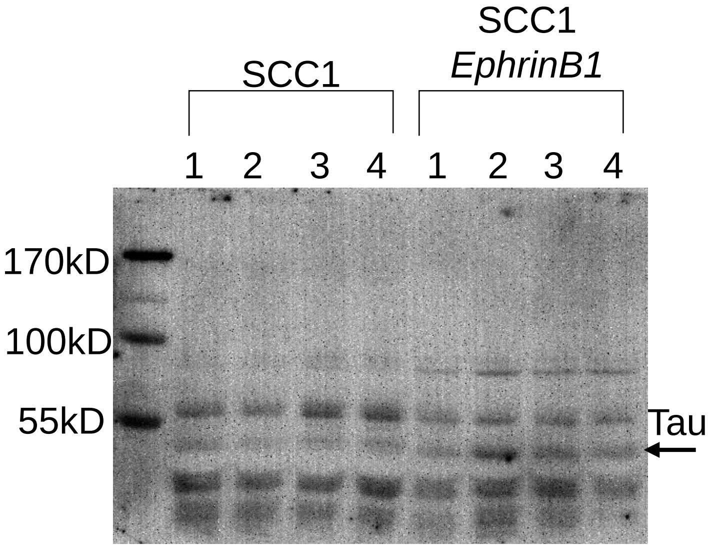

Full western blots for Figure 7 C.

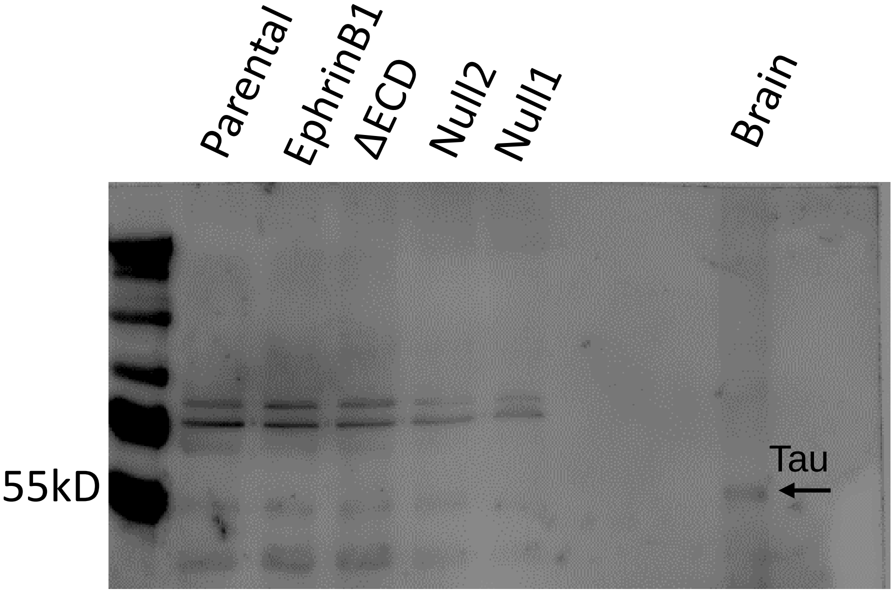

For reference, an additional western in which whole brain lysate was run as a control is included for validation of the Tau band. The samples included with this western blot are whole cell lysates (WCL) from the indicated cell lines.

Supplementary Figure 12
